# The Clinical Significance of the Dense Fine Speckled Immunofluorescence Pattern on HEp-2 Cells for the Diagnosis of Systemic Autoimmune Diseases

**DOI:** 10.1155/2012/494356

**Published:** 2012-12-06

**Authors:** Michael Mahler, Marvin J. Fritzler

**Affiliations:** ^1^INOVA Diagnostics, Inc., 9900 Old Grove Road, San Diego, CA 32131-1638, USA; ^2^Department of Medicine, Faculty of Medicine, University of Calgary, Calgary, AB, Canada T3H 1H7

## Abstract

Antinuclear antibodies (ANAs) are a serological hallmark in the diagnosis of systemic autoimmune rheumatic diseases (SARD). The indirect immunofluorescence (IIF) assay on HEp-2 cells is a commonly used test for the detection of ANA and has been recently recommended as the screening test of choice by a task force of the American College of Rheumatology. However, up to 20% of apparently healthy individuals (HI) have been reported to have a positive IIF ANA test, primarily related to autoantibodies that target the dense fine speckles 70 (DFS70) antigen. Even more important, the DFS IIF pattern has been reported in up to 33% of ANA positive HI, but not in ANA positive SARD sera. Since the intended use of the ANA HEp-2 test is to aid in the diagnosis and classification of SARD, the detection and reporting of anti-DFS70 antibodies and their associated pattern (DFS) as a positive test significantly reduce the specificity and the positive likelihood of the ANA test. This has significant implications for medical management and diagnostic algorithms involving the detection of ANA. Recently, a novel immunoadsorption method has been developed that specifically blocks anti-DFS70 antibodies and, therefore, significantly increases the specificity of the ANA test for SARD. This immunoadsorption method has the potential to overcome a significant limitation of the ANA HEp-2 assay. The present paper summarizes the current knowledge about anti-DFS70 antibodies and their clinical impact on ANA testing.

## 1. History of ANA Testing

The presence of autoantibodies directed against intracellular antigens, especially antinuclear antibodies (ANAs), is a serological hallmark of systemic autoimmune rheumatic diseases (SARD) [1]. In 1958, Friou first described an indirect immunofluorescence (IIF) assay for the detection of antinuclear antibodies (ANA)—which is a historic landmark in the continuing long history of ANA testing in clinical medicine [2]. In most diagnostic laboratories, the ANA test uses HEp-2 tissue culture cells, a cell line which was established in 1952 by Alice E. Moore et al. and derived from tumors that had been produced in irradiated and cortisone treated weanling rats after injection with epidermoid carcinoma tissue obtained from the larynx of a 56-year-old male [3]. The HEp-2 cell—a virtual native protein and nucleic acid array comprised of hundreds if not thousands of potential autoantigens, has been an ideal substrate for the detection of ANA [4]. Over forty years ago and then during the following decades when HEp-2 cells were introduced and used as the IIF substrate of choice, the ANA IIF test using these cells revolutionized the diagnosis of SARD, especially of systemic lupus erythematosus (SLE) and systemic sclerosis. 

In recent years, the IIF assay on HEp-2 cells has been replaced in many laboratories by high throughput and economical screening immunoassays, which incorporate the key SARD autoantibody target antigens into a single assay, on platforms such as ELISA and multiplex assays based on addressable laser bead technology [5]. However, due to a perceived high prevalence of “false negative” results and lack of standardization of innovative test algorithms (i.e., reflex testing) that attended these newer approaches, the American College of Rheumatology (ACR) formed a task force who recommended that the traditional IIF ANA method on HEp-2 cells should remain the screening test of choice [4]. This has resulted in many laboratories moving back to the traditional HEp-2 cell based IIF method as screening test for ANA. 

Coincident with these events, the first digital imaging systems were developed [6, 7] with an advantage of reducing two of the major drawbacks of the ANA IIF method, namely, the subjectivity of human interpretation of IIF results, and the lack of high throughput and automated reading technologies. Nevertheless, several challenges attending the HEp-2 IIF methodology persist [8, 9] and other technologies for ANA detection continue to evolve [10, 11]. 

One of the most important drawbacks of the HEp-2 IIF assay as a screening test is its limited specificity for SARD [9, 12]. Approximately 20% of serum samples from healthy individuals (HI) have been reported to have a positive ANA test [13], the majority of which are reported to be directed to the dense fine speckles 70 (DFS70) antigen [13].

## 2. History and Clinical Association of Anti-DFS70 Antibodies

Anti-DFS70 antibodies were initially identified in a patient with interstitial cystitis [14] but were later associated with various conditions, especially atopic dermatitis [15]. Since their first description, anti-DFS70 antibodies have been found in the sera of patients with a variety of conditions including cancer [16], and even more interestingly in HI [13, 17]. Dellavance et al. evaluated over 10,000 ANA positive samples by HEp-2 IIF followed by a confirmatory immunoblot and reported that anti-DFS70 antibodies were common among ANA-positive individuals with no evidence of SARD and that among autoimmune patients with this autoantibody over a half had evidence of autoimmune thyroiditis [18]. Although the spectrum of clinical associations and the mechanisms of anti-DFS70 induction are still unclear, different research teams have confirmed that anti-DFS70 antibodies are curiously more prevalent in apparently HI than in SARD patients [13, 15]. In addressing the prognostic and long-term outcome of individuals that have anti-DFS70 antibodies, it was recently reported that none of the 40 anti-DFS70 positive HI developed SARD over an average of 4-years of clinical followup [12]. Based on these observations, it has been suggested that the presence of isolated anti-DFS70 antibodies could be used as a biomarker to exclude the diagnosis of SARD, such as SLE [12, 13, 19]. Explanations for the decreased prevalence of anti-DFS70 autoantibodies in SARD patients continue to be unclear, but may relate to concurrent therapeutic, demographic, genetic [20], racial, and/or technological variables.

## 3. IIF Pattern and Cellular Function of DFS70

**Figure 1:**
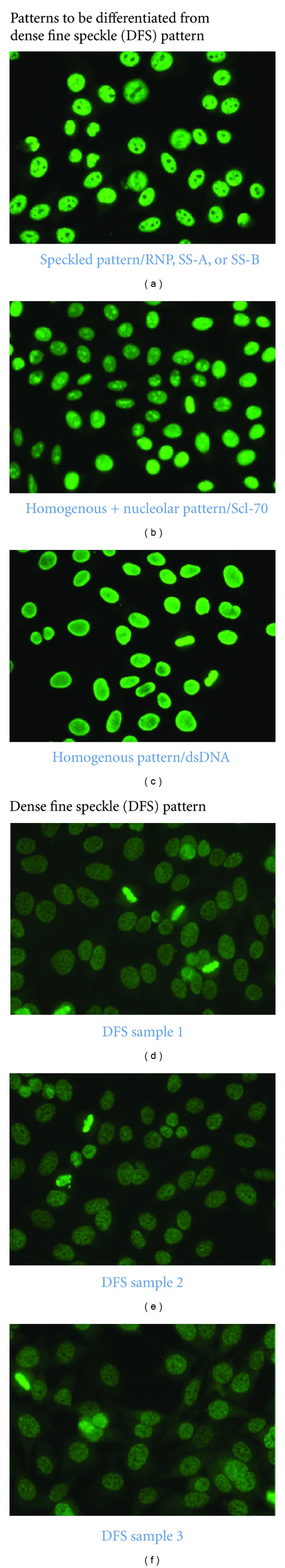
Identification of the dense fine speckled pattern is not always an easy task. The DFS pattern has to be differentiated from (a) speckled patterns generated by autoantibodies against RNP, Ro60 and SS-B (La), (b) from homogeneous patterns generated by anti-Scl-70 antibodies, and from (c) homogenous patterns generated by anti-dsDNA antibodies. DFS patterns generated by three different samples are shown in (d)–(f). Samples (d) and (e) are monospecific anti-DFS70 samples, and sample (f) also contains low titers of antibodies against extractable nuclear antigens (ENA).

The typical IIF staining pattern has been described as DFS that are rather uniformly distributed throughout the interphase nucleus and, most notably, are also localized on metaphase chromosomes (see Figures 1(d)-1(e)) [21]. As with other patterns, the typical DFS pattern can vary depending on the manufacturer source of the HEp-2 slides used as substrate [22]. Since a 70-kDa protein was recognized by immunoblotting, the antigen was initially termed DFS70 but eventually the primary target autoantigen was identified as the lens epithelium derived growth factor (LEDGF) [23] and/or DNA binding transcription coactivator p75 (reviewed in [15]). This protein is highly expressed in prostate tumor tissue [16] and has a number of physiological functions including serving as a cofactor for human immunodeficiency virus replication through interaction with the viral integrase [24].

## 4. Change in ANA Test Referral Pattern

**Figure 2:**
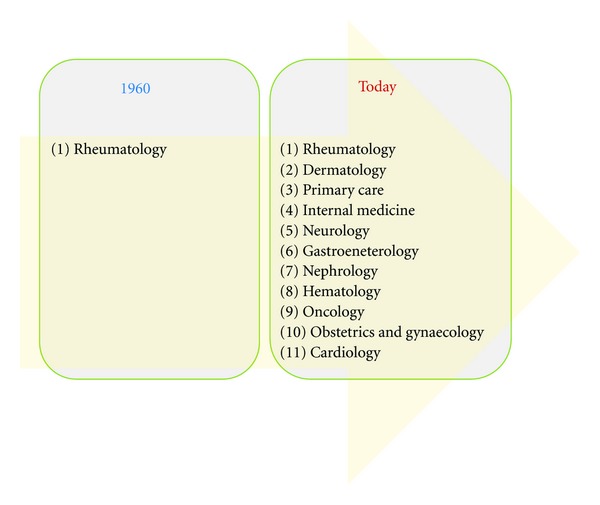
Change in ANA test referral patterns. Historically, when the ANA HEp-2 test became available in the 1960s exclusively rheumatologist ordered the ANA test. With the emerging recognition that many other diseases are associated with ANAs, a broad range of clinical disciplines order the ANA test.

Historically, when the ANA HEp-2 test became available in the 1960s, predominantly rheumatologists and clinical immunologists ordered the ANA test. With the emerging recognition that many other diseases with autoimmune features are also associated with ANAs, a broader range of clinical disciplines (i.e., primary care, dermatology, nephrology, gastroenterology, neurology, oncology, hematology, obstetrics, gynaecology, as well as cardiology) currently order the ANA test (Figure 2). This change in test referral patterns has tremendous consequences for the posttest probability of disease since screening tests with limited specificity (such as IIF ANA) are strongly affected when the pretest probability in a given population decreases.

## 5. Detection of Anti-DFS70 Antibodies

Anti-DFS70 antibodies can be detected by various technologies including IIF [12], immunoblot [12], ELISA [22], addressable laser bead assay (ALBIA, unpublished data), and a novel chemiluminescent assay [25]. Most likely other technologies will be successfully employed such as line immunoassays and lateral flow point of care diagnostics containing purified human DFS70 as one of the antigens. A recent strategy that was developed to assist in the detection of anti-DFS70 antibodies is an immunoadsorption IIF assay [25] as described in the following section.

## 6. Immunoadsorption of Anti-DFS70 Antibodies

**Figure 3:**
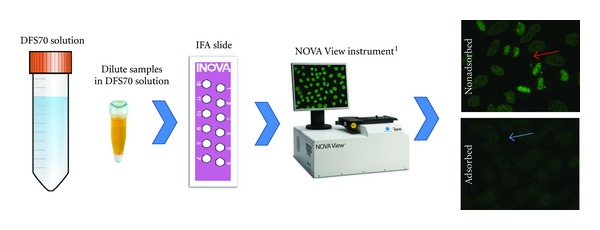
Immunoadsorption of anti-DFS70 antibodies. Serum samples are diluted in sample buffer containing recombinant DFS70 antigen and applied to HEp-2 cells on slides. Following immunodetection using a secondary antibody, indirect immunofluorescence is detected using NOVA View or a conventional microscope. Anti-DFS70 antibodies are specifically blocked.

In a recent study, the DFS IIF pattern was found in 33.1% of ANA positive HI compared to 0.0% of ANA positive patients with SARD (*P* < 0.0001), a result that significantly affects the diagnostic power and efficiency of the IIF assay [12]. Thus, accurate IIF pattern recognition, interpretation, and reporting of results to clinicians are of high importance because it could decrease the necessity of urgent referral of patients with a positive ANA for tertiary care consultation and evaluation [9]. Since the definitive identification of the DFS IIF pattern might be challenging for routine diagnostic laboratories [22] and inaccurate interpretation can have significant consequences, a method that can prevent anti-DFS70 antibodies from binding to their cognate target and producing the DFS pattern was postulated to significantly improve the performance characteristics of ANA by IIF on HEp-2 substrates. Consequently, a novel method allowing for the immunoadsorption of anti-DFS70 antibodies was developed that was meant to yield considerable costsavings by eliminating or reducing unnecessary additional tests (i.e., extractable nuclear antigen profiles or SARD specific autoantibody arrays) [25]. In this approach, patient serum samples are diluted in a sample buffer containing recombinant DFS70 antigen and pipetted onto the HEp-2 cell substrate in wells on glass slides. Following a washing step to remove unbound components, FITC-conjugated antihuman IgG secondary antibody is added. Subsequently, after removing unbound FITC conjugate, the IIF pattern is analyzed under a standard fluorescence microscope or with the NOVA View (INOVA Diagnostics) digital imaging system (see Figure 3). The NOVA View is an inverted microscope that takes pictures of each well, reads common patterns, semiquantifies ANA titers, and through a proprietary algorithm suggests an interpretation of the result. 

## 7. Consequences for ANA Testing: A New Algorithm

**Figure 4:**
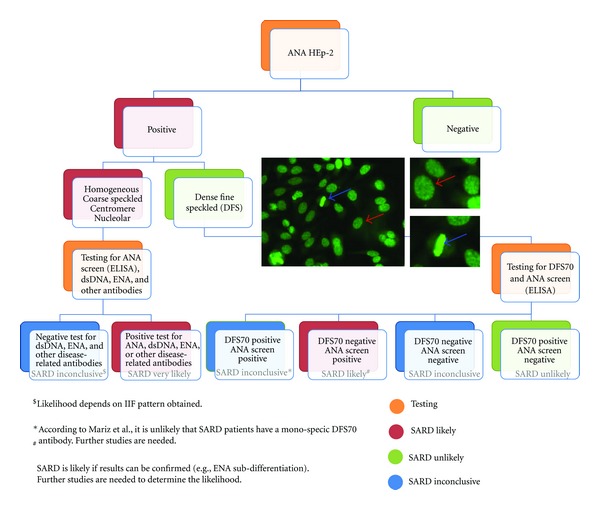
Characteristic staining pattern and suggested test algorithm considering anti-DFS70 antibodies. The characteristic dense fine speckled (DFS) staining pattern of interphase cells is indicated by the red arrow and the strong chromosome staining of metaphase cells by the blue arrow. Samples with a DFS pattern should be tested for anti-DFS70 antibodies by a confirmatory test and by ANA Screen ELISA (QUANTA Lite ANA Screen ELISA) containing various autoantigens. Patients with negative ANA Screen ELISA and positive DFS70 result have a low likelihood for having SARD. Patients with a positive ANA Screen ELISA either in combination with a positive or a negative DFS70 test result have an increase likelihood of having SARD.

In a previous study, 172/21,512 (0.8%) of consecutive serum samples tested for ANA by IIF showed the typical DFS pattern [26] and this was one of the most common IIF patterns observed in the routine clinical diagnostic laboratory. Since the presence of ANA is considered a reliable screening clinical indicators for SARD and are included in the classification criteria for SLE [27], ANA-HEp-2 testing outside a proper clinical framework may yield a sizable portion of ANA-positive individuals with no consistent evidence of SARD. This has the potential to cause undue concern and anxiety in patients, their families, and physicians alike [12], or even lead to unwarranted therapies [28]. This becomes even more crucial with the compelling evidence that autoantibodies appearing in the serum may precede the clinical onset of SARD by many years [29]. As pointed out in a recent article, not all sera demonstrating the DFS pattern are from HI and it remains unclear whether this IIF staining pattern is universally recognized in clinical diagnostic laboratories. The discrimination between DFS and the so-called “quasihomogeneous pattern” might especially be a challenge for routine diagnostic laboratories [9]. This underlines the importance of a better understanding of anti-DFS70 antibodies and the inclusion of testing for anti-DFS70 antibodies into diagnostic algorithms (see Figure 4). A suggestion is that samples with a DFS staining pattern identified by IIF should be tested for anti-DFS70 antibodies using a specific immunoassay and then the test results and the significance of the findings need to be clearly explained to clinicians. 

## Abbreviations

ANA: Antinuclear antibody
DFS: Dense fine speckled
HI: Healthy individuals
IIF: Indirect immunofluorescence
SARD: Systemic autoimmune rheumatic diseases
SLE: Systemic lupus erythematosus.
